# Supplementary material for: Trends in appropriateness of end-of-life care in people with cancer, COPD or with dementia measured with population-level quality indicators
Source: PLoS One. 2023 Feb 1;18(2):e0273997. doi: 10.1371/journal.pone.0273997 (PMC9891500; doi:10.1371/journal.pone.0273997)
Supplement: S1 Table — (DOCX) [file pone.0273997.s001.docx]

**S1 Table: Overview of all measured population characteristics of people dying from cancer in Belgium, from 2010 until 2015.**

| **People who died from cancer in Belgium, 2010-2015 (N = 159,590)** | | | | | | | |
| --- | --- | --- | --- | --- | --- | --- | --- |
|  |  | **2010**  **(N = 26,768)** | **2011**  **(N = 26,820)** | **2012**  **(N = 26,464)** | **2013**  **(N = 26,654)** | **2014**  **(N = 26,391)** | **2015**  **(N = 26,493)** |
| **Average age** |  | 68.2 | 71 | 69.4 | 73.5 | 73.7 | 73.8 |
| **Agecategory** | <65 | 24.6 | 24.4 | 24.4 | 23.3 | 22.8 | 22.9 |
|  | 65-74 | 23.6 | 23.5 | 23.5 | 24.3 | 24.8 | 24.3 |
|  | 75-84 | 35.6 | 35.1 | 34.6 | 34.7 | 34.2 | 34.4 |
|  | >84 | 16.2 | 16.9 | 17.4 | 17.8 | 18.2 | 18.4 |
| **Sex** | Female | 43.6 | 44.2 | 43.5 | 43.9 | 44.1 | 44.1 |
| **Nationality** | Belgian | 95.0 | 94.8 | 95.1 | 95.1 | 95.1 | 94.5 |
| **Householdtype** | Single | 29.3 | 28.9 | 29.8 | 30.0 | 30.2 | 29.9 |
|  | Single parent | 5.0 | 5.0 | 5.3 | 5.0 | 5.3 | 5.3 |
|  | Couple with children | 12.1 | 12.0 | 11.7 | 11.9 | 11.6 | 11.5 |
|  | Couple without children | 44.7 | 44.8 | 44.0 | 44.4 | 44.1 | 44.4 |
|  | Collective  (i.e. nursing home) | 7.1 | 7.2 | 7.1 | 6.9 | 7.0 | 7.2 |
|  | Other | 1.9 | 2.0 | 2.1 | 1.9 | 1.9 | 1.7 |
| **Housing Comfort** | High | 47.2 | 49.1 | 49.6 | 50.4 | 51.5 | 51.6 |
|  | Average | 17.1 | 16.2 | 16.4 | 15.7 | 15.0 | 14.9 |
|  | Low | 26.9 | 26.2 | 25.9 | 26.5 | 26.1 | 26.3 |
|  | None | 8.8 | 8.5 | 8.1 | 7.4 | 7.3 | 7.2 |
| **Highest attained  educational level** | Higher education | . | 11.4 | 12.3 | 12.3 | 13.0 | 13.1 |
|  | Higher secondary | . | 17.2 | 17.2 | 18.0 | 18.4 | 18.7 |
|  | Lower secondary | . | 24.6 | 24.5 | 25.2 | 25.1 | 25.7 |
|  | Primary | . | 28.7 | 28.4 | 27.3 | 27.0 | 25.8 |
|  | None | . | 7.3 | 6.6 | 6.6 | 6.4 | 6.5 |
| **Degree of urbanization  of residence** | Very high | 31.5 | 30.8 | 30.4 | 30.6 | 30.3 | 30.0 |
|  | High | 28.2 | 28.7 | 28.2 | 28.4 | 28.4 | 28.8 |
|  | Average | 26.7 | 26.2 | 26.7 | 26.1 | 26.6 | 26.2 |
|  | Low | 13.6 | 14.1 | 13.9 | 14.0 | 13.8 | 13.9 |
|  | missing | 0.1 | 0.1 | 0.7 | 1.0 | 1.0 | 1.1 |
| **Region** | Flanders | 58.9 | 58.8 | 58.5 | 58.6 | 59.0 | 58.3 |
|  | Wallonia | 33.1 | 33.3 | 33.4 | 33.7 | 33.2 | 34.0 |
|  | Brussels | 8.0 | 7.9 | 8.1 | 7.8 | 7.9 | 7.7 |
| **Net taxable income** | Quintile 1 (highest) | 2.0 | 2.0 | 1.7 | 1.9 | 1.8 | 1.8 |
|  | Quintile 2 | 13.0 | 12.5 | 12.2 | 11.3 | 11.4 | 12.7 |
|  | Quintile 3 | 46.2 | 45.7 | 46.2 | 45.9 | 45.5 | 46.1 |
|  | Quintile 4 | 27.4 | 28.0 | 28.0 | 29.2 | 29.3 | 28.6 |
|  | Quintile 5 (lowest) | 11.4 | 11.8 | 11.9 | 11.8 | 12.0 | 10.9 |
| **Cancertype** | Respiratory | 27.8 | 27.4 | 26.3 | 26.2 | 26.3 | 25.2 |
|  | Digestive tract | 28.9 | 29.0 | 29.1 | 28.5 | 28.5 | 28.4 |
|  | Urinary tract | 6.4 | 6.1 | 6.5 | 6.3 | 6.3 | 6.5 |
|  | Head and neck | 3.2 | 2.9 | 3.2 | 3.4 | 3.4 | 3.4 |
|  | Melanoma | 2.6 | 2.9 | 2.9 | 3.3 | 3.2 | 3.4 |
|  | Breast | 6.9 | 7.2 | 7.6 | 7.5 | 7.6 | 7.6 |
|  | Female genital organs | 5.2 | 5.2 | 5.2 | 4.7 | 4.9 | 4.9 |
|  | Male genital organs | 4.9 | 5.3 | 4.7 | 5.6 | 5.5 | 5.9 |
|  | Other | 13.9 | 13.9 | 14.5 | 14.5 | 14.3 | 14.7 |

*All missings were under 10%, except with education level, since no data are available for 2010.
