# Supplementary material for: Trends in appropriateness of end-of-life care in people with cancer, COPD or with dementia measured with population-level quality indicators
Source: PLoS One. 2023 Feb 1;18(2):e0273997. doi: 10.1371/journal.pone.0273997 (PMC9891500; doi:10.1371/journal.pone.0273997)
Supplement: S2 Table — (DOCX) [file pone.0273997.s002.docx]

**S2 Table: Overview of all measured population characteristics of people dying from COPD in Belgium, from 2010 until 2015.**

| **People who died with COPD in Belgium (N = 37,930)** | | | | | | | |
| --- | --- | --- | --- | --- | --- | --- | --- |
|  |  | **2010**  **(N = 6,878)** | **2011**  **(N = 6,568)** | **2012**  **(N = 6,336)** | **2013**  **(N = 6,274)** | **2014**  **(N = 5,689)** | **2015**  **(N = 6,185)** |
| **Average age** |  | 71.97 | 75.77 | 73.63 | 78.22 | 78.2 | 78.21 |
| **Agecategory** | <65 | 11.72 | 11.81 | 11.16 | 11.44 | 11.55 | 11.62 |
|  | 65-74 | 19.35 | 21.01 | 20.01 | 20.31 | 20.83 | 21.24 |
|  | 75-84 | 44.5 | 42.43 | 41.91 | 41.23 | 40.73 | 40.58 |
|  | >84 | 24.43 | 24.74 | 26.92 | 27.02 | 26.89 | 26.55 |
| **Sex** | Female | 32.68 | 34.61 | 35.63 | 35.58 | 36.23 | 36.7 |
| **Nationality** | Belgian | 93.18 | 93.61 | 93.29 | 93.7 | 93.65 | 93.73 |
| **Living situation** | Single | 31.63 | 31.07 | 32.74 | 31.42 | 31.79 | 31.29 |
|  | Single parent | 5.22 | 4.42 | 4.79 | 4.71 | 4.89 | 4.68 |
|  | Couple with children | 7.58 | 7.91 | 7.2 | 7.53 | 6.74 | 7.12 |
|  | Couple without children | 37.18 | 37.88 | 37.34 | 37.91 | 37.04 | 36.5 |
|  | Collective  (i.e. nursing home) | 15.91 | 16.37 | 15.76 | 16.22 | 17.32 | 18.18 |
|  | Other | 2.48 | 2.35 | 2.16 | 2.19 | 2.22 | 2.23 |
| **Housing Comfort** | High | 35.77 | 36.77 | 37.03 | 38.83 | 38.79 | 39.77 |
|  | Average | 20.61 | 20.35 | 20.44 | 18.6 | 18.99 | 18.73 |
|  | Low | 30.51 | 30.36 | 31.02 | 31.39 | 31.02 | 31.32 |
|  | None | 13.1 | 12.52 | 11.51 | 11.17 | 11.2 | 10.18 |
| **Highest attained  educational level** | Higher education | . | 6.06 | 6.22 | 5.51 | 7.05 | 6.77 |
|  | Higher secondary | . | 11.39 | 11.98 | 12.86 | 12.09 | 12.76 |
|  | Lower secondary | . | 21.44 | 22.03 | 22.68 | 22.85 | 23.31 |
|  | Primary | . | 35.87 | 35.87 | 34.91 | 34.89 | 34.54 |
|  | None | . | 10.72 | 9.78 | 9.99 | 9.44 | 9.09 |
| **Degree of urbanization  of residence** | Very high | 32.6 | 31.82 | 31.91 | 30.3 | 30.74 | 30.88 |
|  | High | 26.32 | 27.41 | 26.72 | 27.72 | 28.92 | 27.53 |
|  | Average | 25.71 | 25.03 | 24.49 | 23.92 | 23.13 | 24.28 |
|  | Low | 15.28 | 15.67 | 16.29 | 16.83 | 15.77 | 16.23 |
| **Region** | Flanders | 49.64 | 48.99 | 46.86 | 47.15 | 46.23 | 48.1 |
|  | Wallonia | 41.68 | 41.99 | 45.25 | 45.07 | 46.17 | 44.47 |
|  | Brussels | 8.67 | 9.02 | 7.89 | 7.78 | 7.6 | 7.44 |
| **Net taxable income** | Quintile 1 (highest) | 2.1 | 1.7 | 1.5 | 1.5 | 1.8 | 1.6 |
|  | Quintile 2 | 9.5 | 9.3 | 8.8 | 8.7 | 7.8 | 9.2 |
|  | Quintile 3 | 57.8 | 56.1 | 58.2 | 57.5 | 55.3 | 58.9 |
|  | Quintile 4 | 25.4 | 27.6 | 25.6 | 27.0 | 28.8 | 25.6 |
|  | Quintile 5 (lowest) | 5.2 | 5.3 | 5.9 | 5.4 | 6.4 | 4.8 |

*All missings were under 10%, except with education level, since no data are available for 2010.
