# Supplementary material for: Trends in appropriateness of end-of-life care in people with cancer, COPD or with dementia measured with population-level quality indicators
Source: PLoS One. 2023 Feb 1;18(2):e0273997. doi: 10.1371/journal.pone.0273997 (PMC9891500; doi:10.1371/journal.pone.0273997)
Supplement: S3 Table — (DOCX) [file pone.0273997.s003.docx]

**S3 Table: Overview of all measured population characteristics of people dying with dementia in Belgium, from 2010 until 2015.**

| **People who died with Dementia in Belgium, 2010-2015 (N = 59,967)** | | | | | | | |
| --- | --- | --- | --- | --- | --- | --- | --- |
|  |  | **2010**  **(N = 10,017)** | **2011**  **(N = 9,891)** | **2012**  **(N = 10,137)** | **2013**  **(N = 9,411)** | **2014**  **(N = 9,882)** | **2015**  **(N = 10,629)** |
| **Average age** |  | 79.48 | 83.46 | 83.25 | 85.99 | 86.19 | 86.6 |
| **Agecategory** | <65 | 1.3 | 1.01 | 0.94 | 0.86 | 0.67 | 0.82 |
|  | 65-74 | 5.06 | 4.64 | 4.52 | 4.47 | 3.95 | 3.87 |
|  | 75-84 | 41.5 | 40.82 | 38.27 | 36.9 | 37.01 | 34.26 |
|  | >84 | 52.14 | 53.52 | 56.26 | 57.77 | 58.37 | 61.05 |
| **Sex** | Female | 65.35 | 65.37 | 65.95 | 66.12 | 66.4 | 65.82 |
| **Nationality** | Belgian | 96.12 | 95.72 | 95.67 | 95.9 | 95.62 | 95.61 |
| **Householdtype** | Single | 20.44 | 20.16 | 20.23 | 19.77 | 19.31 | 18.77 |
|  | Single parent | 3.76 | 4.14 | 4.18 | 4 | 3.8 | 3.65 |
|  | Couple with children | 4.4 | 4.34 | 4.08 | 3.9 | 4.03 | 3.92 |
|  | Couple without children | 27.17 | 26.37 | 25.73 | 25.82 | 25.89 | 24.91 |
|  | Collective  (i.e. nursing home) | 41.87 | 42.79 | 43.65 | 44.23 | 44.97 | 46.53 |
|  | Other | 2.36 | 2.21 | 2.14 | 2.29 | 2.01 | 2.21 |
| **Housing Comfort** | High | 34.54 | 35.13 | 37.33 | 39.01 | 39.62 | 39.98 |
|  | Average | 27.4 | 26.9 | 26.24 | 24.42 | 24.61 | 23.38 |
|  | Low | 25.76 | 25.27 | 25.11 | 25.83 | 25.72 | 26.48 |
|  | None | 12.31 | 12.69 | 11.32 | 10.74 | 10.05 | 10.16 |
| **Highest attained  educational level** | Higher education | . | 6.76 | 6.69 | 7.56 | 7.43 | 7.8 |
|  | Higher secondary | . | 10.48 | 10.97 | 11.57 | 11.5 | 11.97 |
|  | Lower secondary | . | 20.62 | 20.98 | 21.89 | 21.51 | 21.21 |
|  | Primary | . | 37.51 | 38.94 | 36.82 | 37.06 | 37.44 |
|  | None | . | 9.17 | 8.44 | 8.39 | 9.14 | 8.62 |
| **Degree of urbanization  of residence** | Very high | 33.85 | 33.39 | 31.79 | 31.78 | 31.28 | 30.92 |
|  | High | 28.57 | 29.09 | 29.63 | 28.77 | 28.75 | 28.78 |
|  | Average | 24.88 | 24.09 | 24.67 | 24.57 | 24.18 | 25.42 |
|  | Low | 12.61 | 13.41 | 13.16 | 12.97 | 14.25 | 13.2 |
|  | missing | 0.09 | 0.02 | 0.76 | 1.9 | 1.53 | 1.67 |
| **Region** | Flanders | 57.86 | 58.5 | 56.47 | 58.26 | 56.18 | 56.99 |
|  | Wallonia | 32.34 | 32.39 | 34.99 | 33.72 | 35.67 | 35.3 |
|  | Brussels | 9.79 | 9.11 | 8.54 | 8.02 | 8.15 | 7.71 |
| **Net taxable income** | Quintile 1 (highest) | 1.1 | 1.0 | 0.7 | 0.8 | 1.0 | 0.8 |
|  | Quintile 2 | 11.3 | 11.4 | 11.1 | 10.9 | 10.8 | 11.3 |
|  | Quintile 3 | 52.8 | 52.9 | 52.0 | 50.0 | 50.6 | 53.0 |
|  | Quintile 4 | 27.3 | 28.2 | 28.3 | 29.8 | 28.8 | 27.1 |
|  | Quintile 5 (lowest) | 7.5 | 7.7 | 7.93 | 8.62 | 8.8 | 7.8 |

*All missings were under 10%, except with education level, since no data are available for 2010.
