# Supplementary material for: Trends in appropriateness of end-of-life care in people with cancer, COPD or with dementia measured with population-level quality indicators
Source: PLoS One. 2023 Feb 1;18(2):e0273997. doi: 10.1371/journal.pone.0273997 (PMC9891500; doi:10.1371/journal.pone.0273997)
Supplement: S4 Table — (DOCX) [file pone.0273997.s004.docx]

**S4 Table: All indicator scores, not controlled for confounding variables**

| **Indicators appropriate end-of-life care for people dying from cancer** | **2010** | **2011** | **2012** | **2013** | **2014** | **2015** | **Total** |
| --- | --- | --- | --- | --- | --- | --- | --- |
| Opioids | 72.6 | 73.4 | 74.2 | 75.0 | 75.9 | 75.3 | 74.4 |
| Opioids and neuropathic medication | 7.6 | 7.9 | 7.8 | 8.1 | 8.5 | 8.7 | 8.1 |
| Specialist palliative care | 43.9 | 45.4 | 47.1 | 47.7 | 49.4 | 49.4 | 47.1 |
| Official palliative care status | 36.1 | 36.6 | 37.7 | 38.6 | 39.6 | 39.3 | 38.0 |
| Death at home | 29.4 | 29.2 | 29.5 | 29.9 | 29.9 | 29.7 | 29.6 |
| Death at home or in nursing home where resided for at least 180 days | 36.7 | 36.6 | 36.9 | 37.4 | 37.4 | 37.4 | 37.1 |
| Increased number of contact with family physician | 36.6 | 52.2 | 52.4 | 56.0 | 58.8 | 57.7 | 52.2 |
| Average number of family physician contacts | 1.5 | 2.9 | 2.6 | 2.9 | 3.1 | 3.1 | 2.7 |
| Average number of primary caregiver contacts | 8.4 | 9.0 | 8.4 | 8.8 | 9.0 | 9.2 | 8.6 |
| Multidisciplinary oncology consult | 10.5 | 11.8 | 13.2 | 13.6 | 13.9 | 14.4 | 12.9 |
|  | | | | | | | |
| **Indicators inappropriate end-of-life care for people dying from cancer** | **2010** | **2011** | **2012** | **2013** | **2014** | **2015** | **Total** |
| Chemotherapy | 17.6 | 17.1 | 16.9 | 17.0 | 16.7 | 16.7 | 17.0 |
| Cisplatin in old age (Age: 80+) | 0.3 | 0.3 | 0.2 | 0.2 | 0.2 | 0.2 | 0.2 |
| Diagnostic testing (all) | 66.3 | 65.6 | 65.9 | 64.8 | 65.1 | 65.2 | 65.5 |
| Diagnostic testing - medical imaging | 65.0 | 64.4 | 64.6 | 63.5 | 63.7 | 64.0 | 64.2 |
| Diagnostic testing - ECG or pulmonary function testing | 33.7 | 32.5 | 32.4 | 30.9 | 30.5 | 31.4 | 31.9 |
| Portable catheter installed | 0.0 | 0.0 | 1.1 | 1.4 | 1.3 | 1.3 | 0.8 |
| Start taking an antidepressant | 6.7 | 6.9 | 6.5 | 6.0 | 6.4 | 6.3 | 6.5 |
| Surgery | 3.1 | 3.1 | 3.3 | 3.0 | 2.9 | 3.1 | 3.1 |
| Blood transfusion (last 14 days before death) | 3.8 | 4.4 | 4.7 | 4.9 | 4.1 | 4.3 | 4.4 |
| Late initiation of palliative care (of those who received SPC during last 2 years) | 37.3 | 34.9 | 35.5 | 34.3 | 34.6 | 34.6 | 35.2 |
| ED admissions | 33.0 | 32.4 | 33.8 | 34.2 | 35.1 | 35.6 | 34.0 |
| Hospital admissions | 62.6 | 62.0 | 62.0 | 61.0 | 61.9 | 62.1 | 61.9 |
| ICU admissions from nursing home | 2.3 | 2.4 | 2.1 | 2.1 | 2.5 | 2.2 | 2.3 |
|  | | | | | | | |
| **Indicators appropriate end-of-life care for people who died from COPD** | **2010** | **2011** | **2012** | **2013** | **2014** | **2015** | **Total** |
| Opioids | 38.6 | 38.8 | 39.5 | 40.2 | 42.1 | 41.5 | 40.1 |
| Inhalation therapy | 80.4 | 81.7 | 81.7 | 82.1 | 82.0 | 81.3 | 81.5 |
| Specialist palliative care | 8.7 | 8.9 | 10.0 | 11.0 | 11.6 | 13.0 | 10.5 |
| Official palliative care status | 7.2 | 6.9 | 8.2 | 9.9 | 9.7 | 10.1 | 8.7 |
| Death at home | 24.4 | 24.0 | 24.4 | 24.6 | 24.8 | 25.3 | 24.6 |
| Death at home or in nursing home where resided for at least 180 days | 42.2 | 41.8 | 42.2 | 41.8 | 43.0 | 44.2 | 42.5 |
| Increased number of contact with family physician | 34.2 | 43.4 | 45.8 | 50.1 | 53.6 | 54.6 | 46.9 |
| Average number of family physician contacts |  |  |  |  |  |  |  |
| Average number of primary caregiver contacts |  |  |  |  |  |  |  |
|  | | | | | | | |
| **Indicators inappropriate end-of-life care for people who died from COPD** | **2010** | **2011** | **2012** | **2013** | **2014** | **2015** | **Total** |
| Lung volume reduction surgery | 0.2 | 0.1 | 0.1 | 0.1 | 0.1 | 0.0 | 0.1 |
| Endotracheal intubation or tracheotomy | 17.5 | 17.3 | 17.8 | 16.2 | 16.2 | 16.0 | 16.8 |
| Continuous endotracheal intubation | 6.8 | 6.5 | 6.6 | 5.6 | 5.4 | 5.3 | 6.0 |
| Repeated endotracheal intubation | 15.5 | 15.6 | 15.9 | 14.2 | 14.4 | 14.1 | 14.9 |
| Diagnostic testing (all) | 66.3 | 66.6 | 65.9 | 66.4 | 64.7 | 64.9 | 65.8 |
| Diagnostic testing - medical imaging | 64.5 | 64.7 | 64.1 | 64.3 | 62.5 | 63.0 | 63.9 |
| Diagnostic testing - ECG or pulmonary function testing | 46.7 | 46.1 | 45.0 | 43.2 | 41.7 | 42.1 | 44.2 |
| Late physiotherapy | 7.8 | 7.9 | 8.2 | 7.2 | 6.8 | 7.9 | 7.6 |
| Portable catheter installed | 0.0 | 0.0 | 0.1 | 0.1 | 0.1 | 0.0 | 0.0 |
| Coronary or abdominal surgery | 1.5 | 1.6 | 1.5 | 1.4 | 1.4 | 1.2 | 1.4 |
| Reanimation after intubation | 1.8 | 1.9 | 1.7 | 1.7 | 1.6 | 1.5 | 1.7 |
| Start taking an antidepressant | 5.7 | 6.2 | 5.4 | 6.1 | 5.4 | 4.4 | 5.5 |
| Surgery | 1.7 | 1.7 | 2.3 | 1.9 | 2.1 | 1.8 | 1.9 |
| Blood transfusion (last 14 days before death) | 1.4 | 1.4 | 1.5 | 1.1 | 1.3 | 1.2 | 1.3 |
| Late initiation of palliative care | 3.7 | 3.1 | 3.9 | 3.8 | 4.3 | 4.9 | 4.0 |
| ED admissions | 39.0 | 40.5 | 40.5 | 42.8 | 43.0 | 44.5 | 41.7 |
| Hospital admissions | 60.2 | 61.9 | 60.2 | 60.9 | 60.0 | 60.2 | 60.6 |
| ICU admissions | 12.4 | 13.1 | 14.7 | 14.4 | 15.1 | 14.8 | 14.1 |
| ICU admissions from nursing home | 1.6 | 1.4 | 1.5 | 1.5 | 1.9 | 1.6 | 1.6 |
| Hospital death | 54.8 | 55.6 | 54.7 | 54.7 | 53.3 | 51.9 | 54.2 |
|  | | | | | | | |
| **Indicators appropriate end-of-life care for people who died with dementia** | **2010** | **2011** | **2012** | **2013** | **2014** | **2015** | **Total** |
| Opioids and neuropathic medication | 1.8 | 1.8 | 1.7 | 2.3 | 2.2 | 2.2 | 2.0 |
| Specialist palliative care | 8.7 | 9.1 | 9.3 | 9.9 | 10.7 | 9.8 | 9.6 |
| Official palliative care status | 5.7 | 5.5 | 5.4 | 5.8 | 6.6 | 5.8 | 5.8 |
| Death at home | 13.5 | 12.1 | 12.4 | 12.9 | 11.9 | 10.9 | 12.3 |
| Death at home or in nursing home where resided for at least 180 days | 65.1 | 65.4 | 66.6 | 67.8 | 67.7 | 68.4 | 66.8 |
| Increased number of contact with family physician | 24.9 | 46.3 | 46.9 | 63.3 | 72.9 | 74.1 | 54.8 |
| Average number of family physician contacts | 0.7 | 1.8 | 1.8 | 2.9 | 3.6 | 3.6 | 4.4 |
| Average number of primary caregiver contacts | 3.6 | 4.5 | 4.4 | 5.6 | 6.4 | 6.3 | 5.1 |
|  | | | | | | | |
| **Indicators inappropriate end-of-life care for people who died with dementia** | **2010** | **2011** | **2012** | **2013** | **2014** | **2015** | **Total** |
| Diagnostic testing (all) | 39.1 | 38.2 | 37.8 | 36.3 | 35.5 | 36.3 | 37.2 |
| Diagnostic testing - medical imaging | 37.6 | 36.7 | 36.4 | 35.1 | 34.4 | 35.1 | 35.9 |
| Diagnostic testing - ECG or pulmonary function testing | 25.4 | 24.6 | 23.8 | 22.1 | 22.5 | 22.1 | 23.4 |
| Dispension of statins | 5.5 | 5.6 | 5.9 | 6.1 | 5.6 | 6.5 | 5.9 |
| Dispension of gastric protectors | 20.2 | 22.8 | 23.7 | 23.2 | 25.0 | 26.5 | 23.6 |
| Dispension of anti-hypertensives | 42.8 | 43.7 | 43.1 | 42.0 | 42.1 | 43.8 | 42.9 |
| Dispension of calcium vitamin D | 6.1 | 6.6 | 7.3 | 8.0 | 8.5 | 8.6 | 7.5 |
| Dispension of NOAC's or vitamin K antagonists | 35.8 | 36.7 | 36.4 | 36.4 | 36.4 | 38.5 | 36.7 |
| Dispension of prophylactic gout medication | 1.6 | 1.6 | 1.6 | 1.5 | 1.6 | 1.8 | 1.6 |
| Dispension of serotonin reuptake inhibitors | 11.4 | 10.9 | 10.8 | 10.6 | 9.7 | 10.2 | 10.6 |
| Chemotherapy | 0.4 | 0.4 | 0.3 | 0.3 | 0.4 | 0.5 | 0.4 |
| Surgery | 0.6 | 0.8 | 0.9 | 0.7 | 0.6 | 0.8 | 0.7 |
| Blood transfusion (last 14 days before death) | 0.5 | 0.4 | 0.4 | 0.3 | 0.4 | 0.4 | 0.4 |
| Late initiation of palliative care | 4.1 | 4.1 | 4.5 | 4.8 | 4.9 | 4.7 | 4.5 |
| Hospital admissions | 35.2 | 34.7 | 33.7 | 32.3 | 32.7 | 32.4 | 33.5 |
| ED admissions | 23.8 | 24.1 | 24.7 | 24.2 | 24.8 | 25.6 | 24.5 |
| Hospital death | 28.9 | 28.5 | 27.1 | 25.9 | 26.0 | 25.1 | 26.9 |
| ICU admissions | 2.7 | 2.5 | 2.4 | 2.4 | 2.2 | 2.2 | 2.4 |
| ICU admissions from nursing home | 1.1 | 1.1 | 1.1 | 1.3 | 0.9 | 1.0 | 1.1 |
