# Supplementary material for: Trends in appropriateness of end-of-life care in people with cancer, COPD or with dementia measured with population-level quality indicators
Source: PLoS One. 2023 Feb 1;18(2):e0273997. doi: 10.1371/journal.pone.0273997 (PMC9891500; doi:10.1371/journal.pone.0273997)
Supplement: S5 Table — (DOCX) [file pone.0273997.s005.docx]

**S5 Table: The final set of 28 QIs for people with Alzheimer’s disease**

| **Indicator  (brief description)** | **Indicator of appropriate (A) or inappropriate (I) care** | **Numerator  (Number of people who died with Alzheimer’s disease who*)** | **Denominator**  **(*Number of people who died with Alzheimer's disease)** |
| --- | --- | --- | --- |
| **Domain**: aggressiveness of care** | | | |
| **Tube feeding or intravenous feeding^†§^** | **I** | ***received tube feeding or intravenous feeding in the last month prior to death** | ***** |
| Reanimation^‡§^ | I | *were reanimated in the last 2 weeks prior to death | * |
| **Blood transfusion^‡§^** | **I** | ***received blood transfusion in the last month prior to death** | ***** |
| **Diagnostic testing^‡§^** | **I** | ***had diagnostic testing (spirometry OR radiography OR blood drawn OR electrocardiogram) in the last month prior to death** | ***** |
| Neurologist visit^‡\\^ | I | *received treatment from a neurologist in the last month prior to death | * |
| **Port-a-cath installment^‡§^** | **I** | ***had a port-a-cath installed in the last 2 weeks prior to death** | ***** |
| **Surgery^‡§^** | **I** | ***received surgery in the last [6,3, 1] months prior to death** | ***** |
| Statins^‡§^ | I | *received statins and did not have declining statin use in the last [12,6,1] months prior to death | * and received statins |
| Gastric protectors^‡\\^ | I | *received two or more prescriptions of gastric protectors in the last 6 months prior to death (ie prescription until death) | * |
| Anti-hypertensives^‡§^ | I | *received antihypertensives in the last [6,3,1] months prior to death | * |
| Calcium vitamin D^‡§^ | I | *received calcium or vitamin D in the last [6,3,1] months prior to death | * |
| NOACs or vitamin K antagonists^‡\\^ | I | *received a prescriptions for novel oral anticoagulants OR vitamin K antagonists in the last 3 months prior to death (i.e. prescription until death) | * |
| Prophylactic gout medication^‡\\^ | I | *received a prescriptions for prophylactic gout medication in the last 3 months prior to death | * |
| Serotonin reuptake inhibitors^‡\\^ | I | *received serotonin reuptake inhibitors in the last 3 months prior to death | * |
| Chemotherapy for cancer patients with Alzheimer’s disease^‡\\^ | I | * had a cancer diagnosis and received chemotherapy in the [12, 6] months prior to death | * and had a cancer diagnosis |
| **Domain: Pain and symptom treatment** | | | |
| Morphine and neuropathic medication^†§^ | A | *received neuropathic medication when receiving morphine in the last 2 years prior to death | * |
| **Domain: Palliative care** | | | |
| **Specialized palliative care^†§^** | **A** | ***received specialized palliative care (Hospital palliative unit OR palliative daycare centre OR multidisciplinary home care) in the last 2 years prior to death** | ***** |
| **Official palliative care status^†§^** | **A** | ***received official palliative care status, enabling financial government support for palliative care at any point prior to death** | ***** |
| **Late initiation of palliative care ^†§^** | **I** | ***had a first referral to specialized palliative care OR received the official palliative statute in the last week before death** | ***** |
| **Domain: Place of treatment and place of death** | | | |
| **Hospital admissions^†§^** | **I** | ***had one or more hospital admissions in the last [6, 3, 1] month/s prior to death** | ***** |
| ICU admissions^‡§^ | I | *had one or more admissions to the intensive care unit in the last month prior to death | * |
| **ED admissions^†§^** | **I** | ***had one or more emergency department visit/s in the last [6, 3, 1] month/s prior to death** | ***** |
| Died in hospital^†§^ | I | *died in hospital | * |
| **Home death^†§^** | **A** | ***died at home** | ***** |
| Home death or death in nursing home of residence ^†§^ | A | *lived and died in a nursing home OR who died at home | * |
| **Domain: Coordination and continuity of care** | | | |
| **GP contact^†§^** | **A** | ***an increase in average number of contacts with a family physician in the last month prior to death compared to the previous 23 months** | ***** |
| **Primary caregiver contact^†§^** | **A** | **Sum of number of contacts with a family physician or other primary care professional in the last 3 months prior to death** | ***** |
| **ICU admissions from nursing home^‡§^** | **I** | ***lived in a nursing home and had one or more intensive care unit admissions in the last month prior to death** | ***and lived in a nursing home** |

^†^Indicator from literature, ^‡^Indicator from expert interviews, ^§^Accepted in phase 3a scoring round, ^¶^Accepted in phase 3b plenary discussion, ^\\^Adapted and accepted in phase 3b plenary discussion.

**Subdivision in domains was not part of the original methodology, but added later to facilitate interpretation and was based on existing classification of quality domains in end-of-life-care^43^

Bold denotes indicators that are common across all three pathologies (cancer, COPD, Alzheimer’s).
