# Supplementary material for: Trends in appropriateness of end-of-life care in people with cancer, COPD or with dementia measured with population-level quality indicators
Source: PLoS One. 2023 Feb 1;18(2):e0273997. doi: 10.1371/journal.pone.0273997 (PMC9891500; doi:10.1371/journal.pone.0273997)
Supplement: S6 Table — (DOCX) [file pone.0273997.s006.docx]

**S6 Table: The final set of 26 QIs for people with cancer**

| **Indicator  (brief description)** | **Indicator of appropriate (A) or inappropriate (I) care** | **Numerator  (Number of people who died with cancer who*)** | **Denominator**  **(*Number of people who died with cancer)** |
| --- | --- | --- | --- |
| **Domain**: aggressiveness of care** | | | |
| **Tube feeding or intravenous feeding^†§^** | **I** | ***received tube feeding or intravenous feeding in the last month prior to death (excluding people with gatro-intestinal cancer)** | ***** |
| **Blood Transfusion^‡\\^** | **I** | ***received blood transfusion in the last month before death (for people with a hematologic condition in the last 2 weeks)** | ***** |
| **Diagnostic Testing^‡§^** | **I** | ***had diagnostic testing (spirometry OR radiography OR blood drawn OR electrocardiogram) in the last month prior to death** | ***** |
| **Port-a-cath installment^‡§^** | **I** | ***had a port-a-cath installed in the last 2 weeks prior to death** | ***** |
| **Surgery^‡§^** | **I** | ***received surgery in the last [6,3, 1] month/s prior to death** | ***** |
| Chemotherapy ^†\\^ | I | *received 1 or more chemotherapy treatments in the last month prior to death | * |
| Chemotherapy interval^†\\^ | I | Number of days between last chemotherapy treatment and death | * |
| Chemotherapy new Line^†§^ | I | *had a new chemotherapy line initiated in the last 3 months prior to death | * |
|  |  |  |  |
| Cisplatin in old age^‡§^ | I | *received Cisplatin and were age 80 or older | *and were age 80 or older |
| New anti-depressant^‡\\^ | I | *had initiation of a new anti-depressant treatment in the last 2 months prior to death | * |
| **Domain: Pain and symptom treatment** | | | |
| Opioids^†§^ | A | *received opioids in the last [6,3, 1] months prior to death | * |
| Morphine and neuropathic medication^†§^ | A | *received neuropathic medication when receiving morphine in the last 2 years prior to death | * |
| Anti-emetics with chemotherapy^†§^ | A | *received strong anti-emetics when receiving chemotherapy in the last 2 years prior to death | * and received chemotherapy |
| Radiotherapy with Bone Metastases^†§^ | A | *had bone metastasis and received radiation therapy in the last 2 years prior to death | *and had bone metastasis |
| Radiotherapy with small cell lung cancer^‡§^ | A | *died with small cell lung cancer and received antalgic radiotherapy in the last 2 years prior to death | *and had small cell lung cancer |
| **Domain: Palliative care** | | | |
| **Specialized palliative care^†§^** | **A** | ***received specialized palliative care (Hospital palliative unit OR palliative daycare center OR multidisciplinary home care) in the last 2 years prior to death** | ***** |
| **Official palliative care status^†§^** | **A** | ***received official palliative care status, enabling financial government support for palliative care at any point prior to death** | ***** |
| **Late initiation of palliative care ^†¶^** | **I** | ***had a first referral to specialized palliative care OR received official palliative status in the last week before death** | ***** |
| **Domain: Place of treatment and place of death** | | | |
| **Hospital admissions^†§^** | **I** | ***had one or more hospital admissions in the last [6, 3, 1] months prior to death** | ***** |
| **ICU admissions from nursing home^‡§^** | **A** | *** lived in a nursing home and had one or more intensive care unit admissions in the last [6, 3, 1] months prior to death** | ***and lived in a nursing home** |
| **ED admissions^†§^** | **I** | ***had one or more emergency hospital visits in the last [6, 3, 1] months prior to death** | ***** |
| **Home death^†§^** | **A** | ***died at home** | ***** |
| Death in nursing home of residence ^†\\^ | A | *died in the nursing home where they lived | *and lived in a nursing home |
| **Domain: Coordination and continuity of care** | | | |
| **GP contact^†§^** | **A** | *** had an increase in average number of contacts with a family physician in the last month prior to death compared to the previous 23 months** | ***** |
| **Primary caregiver contact^†§^** | **A** | **Total number of contacts with a family physician or other primary care professional in the last 3 months prior to death** | ***** |
| Multi-disciplinary Oncologic Consult^‡§^ | A | *had a Multidisciplinary Medical Consult | * |

^†^Indicator from literature, ^‡^Indicator from expert interviews, ^§^Accepted in phase 3a scoring round, ^¶^Accepted in phase 3b plenary discussion, ^\\^Adapted and accepted in phase 3b plenary discussion.

**Subdivision in domains was not part of the original methodology, but added later to facilitate interpretation and was based on existing classification of quality domains in end-of-life-care^43^

Bold denotes indicators that are common across all three pathologies (cancer, COPD, Alzheimer’s).
