# Supplementary material for: Trends in appropriateness of end-of-life care in people with cancer, COPD or with dementia measured with population-level quality indicators
Source: PLoS One. 2023 Feb 1;18(2):e0273997. doi: 10.1371/journal.pone.0273997 (PMC9891500; doi:10.1371/journal.pone.0273997)
Supplement: S7 Table — (DOCX) [file pone.0273997.s007.docx]

**S7 Table: The final set of 27 QIs for people with COPD**

| **Indicator  (brief description)** | **Indicator of appropriate (A) or inappropriate (I) care** | **Numerator**  **(Number of people who died with COPD who*)** | **Denominator (*Number of people who died with COPD)** |
| --- | --- | --- | --- |
| **Domain: aggressiveness of care** | | | |
| **Tube feeding or intravenous feeding ^†§^** | **I** | ***received tube feeding or intravenous feeding in the last month prior to death** | ***** |
| Endotracheal intubation or tracheotomy^†§^ | I | *received endotracheal intubation or tracheotomy in the last [12, 6, 3, 1] months prior to death | * |
| Continuous endotracheal intubation^†§^ | I | *received continuous endotracheal intubation for 5 days or more in the last [12, 6, 3, 1] months prior to death | * |
| Repeated intubation^‡§^ | I | *received intubation 2 or more times in the last [12, 6, 3, 1] months prior to death | * |
| Reanimation after intubation^‡§^ | I | *were reanimated after intubation in the last week prior to death | * |
| Late physiotherapy^‡¶^ | I | *started physiotherapy treatment in the last 2 weeks prior to death | * |
| **Blood transfusion^‡\\^** | **I** | ***received blood transfusion in the last month prior to death** | ***** |
| **Port-a-cath installment^‡§^** | **I** | ***had a port-a-cath installed in the last 2 weeks prior to death** | ***** |
| Lung volume reduction surgery^†§^ | I | *received Lung Volume Reduction Surgery in the last 3 months prior to death | * |
| Coronary or abdominal surgery^‡§^ | I | *received coronary or abdominal surgery in the last 3 months prior to death | * |
| **Surgery^‡§^** | **I** | ***received surgery in the last [6, 3, 1] months prior to death** | ***** |
| **Diagnostic testing^‡§^** | **I** | ***had diagnostic testing (spirometry OR radiography OR blood drawn OR electrocardiogram) in the last month prior to death** | ***** |
| Starting Antidepressants^‡\\^ | I | *received antidepressants in the last month prior to death and did not receive antidepressants before | * |
| **Domain: Pain and symptom treatment** | | | |
| Opioids^†§^ | A | *received opioids in the last [6,3, 1] months prior to death | * |
| Inhalation therapy^‡§^ | A | *received inhalation corticosteroids OR anticholinergics OR Beta-2-memetics in the last [6, 3, 1] months prior to death | * |
| **Domain: Palliative care** | | | |
| **Specialized palliative care^†§^** | **A** | ***received specialized palliative care (hospital palliative unit OR palliative daycare centre OR multidisciplinary home care) in the last 2 years prior to death** | ***** |
| **Official palliative care status^†§^** | **A** | *** received official palliative care status, enabling financial government support for palliative care at any point prior to death** | ***** |
| **Late initiation of palliative care ^†¶^** | **I** | *** had a first referral to specialized palliative care OR received official palliative status during the last week before death** | ***** |
| **Domain: Place of treatment and place of death** | | | |
| **Hospital admissions^†¶^** | **I** | *** had one or more hospital admission/s in the last [6, 3, 1 }months prior to death** | ***** |
| ICU admissions^‡§^ | I | * had one or more admissions to the intensive care unit in the last month prior to death | * |
| **ICU admissions from nursing home^‡§^** | **I** | ***lived in a nursing home and had 1 or more ICU visits in the last month prior to death** | ***and lived in a nursing home** |
| **ED admissions^†\\^** | **I** | *** had one or more emergency hospital visits in the last [6, 3, 1 }months prior to death** | ***** |
| Hospital death^†¶^ | I | *died in hospital | * |
| **Home death^†§^** | **A** | ***died at home** | ***** |
| Death in nursing home of residence ^†§^ | A | *lived and died in a nursing home | *and lived in a nursing home |
| **Domain: Coordination and continuity of care** | | | |
| **GP contact^†§^** | **A** | ***had an increase in average number of contacts with a family physician in the last month prior to death compared to the previous 23 months** | ***** |
| **Primary caregiver contact ^†§^** | **A** | **Sum of number of contacts with a family physician or other primary care professional in the last 3 months prior to death** | ***** |

^†^Indicator from literature, ^‡^Indicator from expert interviews, ^§^Accepted in phase 3a scoring round, ^¶^Accepted in phase 3b plenary discussion, ^\\^Adapted and accepted in phase 3b plenary discussion. **Subdivision in domains was not part of the original methodology, but added later to facilitate interpretation and was based on existing classification of quality domains in end-of-life-care^43^

Bold denotes indicators that are common across all three pathologies (cancer, COPD, Alzheimer’s).
