# Supplementary material for: Trends in appropriateness of end-of-life care in people with cancer, COPD or with dementia measured with population-level quality indicators
Source: PLoS One. 2023 Feb 1;18(2):e0273997. doi: 10.1371/journal.pone.0273997 (PMC9891500; doi:10.1371/journal.pone.0273997)
Supplement: Attachment — Submitted filename: Response to reviewers.docx [file pone.0273997.s008.docx]

Trends in appropriateness of end-of-life care in people with cancer, COPD or with dementia measured with population-level quality indicators

**PONE-D-21-14989 - Reply to reviewer’s comments**

This document lists all reviewer's responses and comments, with answers and changes to the text, if applicable. All line numbers are referring to the manuscript with tracked changes.

| **Comments by the Academic editor** | | | | |
| --- | --- | --- | --- | --- |
| **Editor comment** | **Response** | | **Changes to the manuscript** | |
| 1. Please review and respond to the peer reviewer comments. | We reviewed and responded to all peer reviewer comments (see below). | | N/A | |
| 2. Please review and revise the study reporting using guidance for observational studies - STROBE and extension for routine data RECORD. Please include completed checklists as supplementary files, referenced in the methods. | We reviewed the study reporting, using the STROBE and RECORD guidance. We included the completed checklists. | | We changed line 306 to: "We report our results following the STROBE and RECORD guidelines" | |
| 3. Please also proof read carefully to correct all typos. | We corrected all typos carefully. | | Several small corrections throughout the manuscript. | |
| 4. Please ensure that your manuscript meets PLOS ONE's style requirements, including those for file naming. The PLOS ONE style templates can be found at | We adapted the manuscript to the style requirements. | | We changed heading layout and removed italic layout, we removed figures from the manuscript and saved them in different .tiff files. We changed references to figures within the manuscript. We changed reference style. We changed the author descriptions and contact details. | |
| 5. In ethics statement in the manuscript and in the online submission form, please provide additional information about the database used in your retrospective study. Specifically, please ensure that you have discussed whether all data were fully anonymized before you accessed them and/or whether the IRB or ethics committee waived the requirement for informed consent. If patients provided informed written consent to have their data used in research, please include this information. | We added a sentence to the ethics statement. | | We added this sentence to lines 313-314: "Only anonymized data were used and small cell analysis was performed to prevent re-identification." | |
| 6. In your Data Availability statement, you have not specified where the minimal data set underlying the results described in your manuscript can be found. | We will elaborate in the data availability statement why the data cannot be shared. | | N/A | |
| 7. Please include captions for your Supporting Information files at the end of your manuscript, and update any in-text citations to match accordingly. | We updated all captions for Supporting Information files and updated in-text citations. | | Various changes throughout the document. | |
| 8. Please remove any funding-related text from the manuscript and let us know how you would like to update your Funding Statement. | We removed any funding-related text from the manuscript and will provide the information to update the Funding Statement. | | We removed the funding statement lines 555-558 in the manuscript. | |
| 9. We note that you have included the phrase “data not shown” in your manuscript. Unfortunately, this does not meet our data sharing requirements. | We changed the results to be presented accordingly, only mentioning results also presented in the table below. | | We changed lines 324-329 to: "The mean age at death was 68.2 in people with cancer, 72.0 in people with COPD and 79.5 in people with dementia. The mean age at death in 2015 was 73.8 in people with cancer, 78.2 in people with COPD and 86.6 in people with dementia." | |
| 10. Your abstract cannot contain citations. Please only include citations in the body text of the manuscript, and ensure that they remain in ascending numerical order on first mention. | We changed the abstract accordingly. | | We removed the numbers between brackets in the abstracts. (1), (2) and (3). | |
| 11. Your ethics statement should only appear in the Methods section of your manuscript. If your ethics statement is written in any section besides the Methods, please move it to the Methods section and delete it from any other section. Please ensure that your ethics statement is included in your manuscript, as the ethics statement entered into the online submission form will not be published alongside your manuscript. | We moved the conflict of interest statement, the reporting and ethics statement and the data-sharing statement to the methods section. | | We moved the conflict of interest statement, the reporting and ethics statement and the data-sharing statement from the discussion section to the methods section. | |
| 12. We note that you have referenced (ie. Bewick et al. [5]) which has currently not yet been accepted for publication. Please remove this from your References and amend this to state in the body of your manuscript: (ie “Bewick et al. [Unpublished]”) as detailed online in our guide for authors  http://journals.plos.org/plosone/s/submission-guidelines#loc-reference-style | Meanwhile, the reference paper has been accepted for publication. We updated the references list accordingly. | | N/A | |
| **Reviewer's Responses to Questions** | | | | |
| **Question** | | **Answers** | | **Reply** |
| 1. Is the manuscript technically sound, and do the data support the conclusions? | | Reviewer #1: Yes  Reviewer #2: Yes  Reviewer #3: Yes | | Thank you. |
| 2. Has the statistical analysis been performed appropriately and rigorously? | | Reviewer #1: I Don't Know  Reviewer #2: Yes  Reviewer #3: Yes | | Thank you. |
| 3. Have the authors made all data underlying the findings in their manuscript fully available? | | Reviewer #1: Yes  Reviewer #2: No  Reviewer #3: Yes | | Unfortunately, the data are not publicly available and due to privacy regulations, cannot be shared by the authors. This is mentioned in the data statement at the end of the manuscript. |
| 4. Is the manuscript presented in an intelligible fashion and written in standard English? | | Reviewer #1: Yes  Reviewer #2: Yes  Reviewer #3: Yes | | Thank you. |
| **Review Comments to the Author** | | | | |
| **Reviewer comment** | | **Response** | | **Changes to the manuscript** |
| **Reviewer #1** | | | | |
| 1.1 Thank you very much for the opportunity to review this manuscript. The topic is important and the paper strong by its use of linked population-data from eight datasets. My one concern is that 5 years seems a short time period for a trend analysis. That said I enjoyed reading the manuscript and feel it has important findings. Some more specific comments are as follows: | | Thank you for reviewing our manuscript. We agree 6 (rather than 5) years is a relatively short period for a trend analysis. We would like to repeat this research in a larger timeframe in the future, albeit with a smaller set of quality indicators, as the large set we used in this study strongly inflates the volume of data needed when measured over more years. | | N/A |
| 1.2 Abstract: The results section is a little wordy - perhaps including some data would improve this. I'm not sure the conclusion necessarily supports the rest of the abstract. It is the first time generalist palliative care, indicators of inappropriate care and medication is mentioned. Perhaps consider re-writing. | | Thank you, we have rephrased the abstract accordingly, removing some text from the results paragraph and changing the conclusion. Changes are also in line with comment 2.2. (see below) | | Lines 67-68, we removed: “, all in the last 30 days.”  Line 74-79 changed to: “To increase the appropriateness of end-of-life care, our results suggest that governments and practitioners should focus on early initiation of palliative care, as well as prevention of unnecessary care transitions and medication.” |
| 1.3 Introduction: Clear. Consider including the following relevant reference: Henson LA, Gomes B, Koffman J, Daveson BA, Higginson IJ and Wei G. Factors associated with aggressive end of life cancer care. Supportive Care in Cancer 2016; 24(3):1079-1089. | | We added the suggested reference as reference nr 10. | | (see line 98) |
| 1.4 The reference from Earle et al reports data from the 1990's which is quite out of date now. Consider removing. | | We prefer to keep this reference, as we consider it to still be relevant, despite its age. | | N/A |
| 1.5 Methods: Clear and detailed. Suggest statistical review. | | Thank you. | | N/A |
| 1.6 Results: Typo in first sentence with writing of 1ste. | | Thank you, we corrected the typo. | | See line 322. |
| 1.7 I don't find figures 1, 2 and 3 useful. If anything they look like not much has changed over the time period. Consider re-drafting and highlighting a few of the most interesting results rather than a figure with all the findings. Then the scale could be changed so the difference over time more easily visualised. | | Thank you, we considered highlighting a smaller selection of results, rather than showing the figure with all indicators. However, we think it is equally important to visualize that some indicators have hardly changed, despite government and practitioner initiatives to improve the quality of care. This is congruent with the remark of reviewer 3 (comment 3.1). | | N/A |
| 1.8 Discussion: Clear and interesting. Limitations acknowledged. | | Thank you. | | N/A |
| **Reviewer # 2** | | | | |
| 2.1 Thank you for the opportunity to review this interesting paper. The article explores an important topic and presents the results of an original research. Understanding changes/shifts in the quality of care provided to patients at the end of life at a population level is key to improve services and, as the ultimate aim, to improve patients’ quality of life. The article is well written, and the methods are clear and rigorous. An important strength of this study is the use of a population-based dataset, of almost the entire population of Belgium. I think some sections and tables could be reported differently to improve clarity.  Find below some specific comments and suggestions by section. | | Thank you for complimenting our manuscript. | | N/A |
| 2.2 Abstract  Introduction: I think the introduction could be clearer in terms of the importance of measuring the appropriateness of care and make the link between appropriateness as a measure of quality of care, and the importance this can have for improving services. | | Thank you for this suggestion. We changed the introduction of the abstract. Changes are also in line with comment 1.2. | | Lines 42-46 changed to: "Measuring changes in the quality of end-of-life care provided to patients with advanced illness such as cancer, COPD or dementia at a population level in terms of the appropriateness of care can help governments and practitioners improve service delivery and patients’ quality of life. However, an assessment of a possible shift in appropriateness of end-of-life care across the population is lacking." |
| 2.3 From my point of view, the emphasis in the conclusion should be put on indicators governments should be focusing on, based on the lack of ‘improvement’ or ‘worsening’, rather than interventions. | | Thank you for this remark. We changed the abstract conclusion accordingly. | | Line 71-77 changed to: “To increase the appropriateness of end-of-life care, our results suggest that governments and practitioners should focus on early initiation of palliative care, as well as prevention of unnecessary care transitions and medication.” |
| 2.4 Introduction:  In the second paragraph, the sentence ‘In contrast, research shows there has mainly been an increase in indicators…’ reads as if the numbers of indicators have increased. Perhaps it would be clearer if you said there has been an increase in overaggressive care measured through these quality indicators...? | | We changed the text accordingly to clarify. | | Line 98-101 changed to: “In contrast, research shows there has mainly been an increase in aggressive end-of-life treatment in western countries.” |
| 2.5 In the third paragraph, you mention the efforts and interest from governments towards measuring the appropriateness/inappropriateness of end-of-life care, but I would suggest giving more emphasis on the relevance of this. Evaluating the quality of care through appropriateness/inappropriateness indicators can help improve service delivery and has the ultimate purpose of improving the quality of life for patients. | | We agree that more emphasis could be placed on the relevance of measuring the appropriateness/inappropriateness to improve the quality of end-of-life care. We have therefore added a sentence to the revised manuscript. | | We changed line 132-136 to: “While measuring and understanding changes in the quality of care provided to patients at the end-of-life at a population level through appropriateness and inappropriateness indicators can help governments and practitioners improve service delivery and patients’ quality of life, a comprehensive …“ |
| 2.6 Methods  I think you should be clearer (here or in the introduction) about the reasons to include cancer, dementia and COPD patients in the analysis and not other terminal conditions. I agree the three conditions included are important and prevalent and they clearly could benefit from a palliative care approach at the end of life. But there are other conditions too. Does that follow a pragmatical approach in terms of the data available or there is also an argument about the conditions that have been target by the government in Belgium? | | Thank you for this remark. The answer is twofold: cancer, COPD and dementia represent three distinct disease trajectories near the end of life identified by Murray et al.^1^ We added a sentence and a reference to clarify this. Also, as we mention in the introduction, there have indeed been effort to increase quality of end-of-life care in these three pathology groups in Belgium: (line 110-113) While traditionally, these efforts focus on people with cancer, efforts have also been made to increase access to palliative care and promote advance care planning for people with dementia and COPD. | | Lines 157-161 we added: “Cancer, COPD and dementia represent the three distinct disease trajectories for people with chronic progressive illness, identified by Murray et al.^1^” |
| 2.7 It is not very clear why you decided to change the period before death for some indicators to the last 30 days. I can see that approach facilitates comparison between indicators and conditions. But I also assume there was a reason for the period when the indicator was defined. For instance, the appropriateness of a hospital admission might be different if you consider the last 6 months vs the last 30 days. | | The reviewer is correct that we measured all indicators in the last 30 days of life to facilitate comparison between indicators and conditions.  However, we mention this in the manuscript. We would like to refer the reviewer to lines 229-236 where we stated that “The quality indicators measure the prevalence of specific healthcare interventions in a specified period before death (e.g. 7 days, 14 days, 30 days, 180 days), within the specific population they were developed for. For the purpose of this trend study all indicators were measured for the period of 30 days before death, except when specific indicators were validated by the expert panel for a shorter time period only (eg 14 days), or time before death was irrelevant for the quality indicator.” | | N/A |
| 2.8 A table with the list of the different indicators used by condition with their definition would be useful (Perhaps is in the supplementary material?). What an ‘Official palliative care status’ means for instance. It would be easier to follow if you can identify/specify initially which indicators are considered inappropriate or appropriate care. It might not be that intuitive for some readers. | | Thank you for this comment. The detailed description of each indicator is available in a previous publication. As we already have 4 supplementary tables, we prefer to refer to the previous publication and its supplemental files. (reference 9: ^2^)  However, we include the tables as attachment to this document, to be added by the editor as they see fit. | | N/A |
| 2.9 In the ‘Risk adjustment and trend analysis’ section, you mention the variables used as confounders. I think one important variable to consider is the level of multimorbidity. Having multiple comorbidities has been consistently associated with higher use of community health care services and hospital admissions (Browne et al, BMJ Open 2017; Soley-Bori et al, Br J Gen Pract. 2021), and the proportion of adults with two or more comorbidities has increased in the last decade in most European countries. (Souza et al, BMC Public Health. 2021). This is particularly relevant for indicators related to healthcare service use. Is it possible that the variables ‘being entitle to a higher degree of reimbursement due to lower degree of self-reliance’ and ‘being officially recognized as having high care needs’ are representing a measure of multimorbidity or complex healthcare needs in this population? It would be useful to discuss that or give more information about those two variables in order to understand to what extend multimorbidity is a factor that has been accounted for in the analysis. | | We agree with the reviewer that multimorbidity is a very important factor associated with health care service use.  As the reviewer mentions, both variables ‘being entitled to a higher degree of reimbursement due to lower degree of self-reliance’ and ‘being officially recognized as having high care needs’, are sometimes used as proxy measures of care dependence, which is known to be linked with multimorbidity^3^.  We have added a sentence to the risk adjustment and trend analysis paragraph where we explain this. We also added a reference. | | Lines 260-273 changed to: "The following risk factors were identified as relevant and measurable with the current dataset: (…) being officially recognized as having high care needs, and being entitled to a higher degree of reimbursement due to lower degree of self-reliance. These last two variables represent measures of care dependence, which is known to be linked with multimorbidity^3^, and they are based on receiving any of the allowances for a highly care-dependent person as judged by the GP, for a person with handicap, or for an elderly person." |
| 2.10 Results  Typo in the first paragraph of ‘cohort characteristics’ ‘1ste January 2010’ (should be 1st) | | Thank you, we corrected the typo. | | See Results, line 322. |
| 2.11 In table 1, I would suggest adding columns represent %s to be clear (except for the mean for age). It would be useful to add missing values in this table if present. | | Thank you for this suggestion. We added a footnote to the table. | | As a footnote to the table, we added (lines 333-334): “^a^Columns represent column percentages, except for the average age. All missing values are below 10%.” |
| 2.12 In the ‘Risk adjusted differences…’ section, I would suggest using subheading to make it easier to follow. Perhaps by condition or by appropriateness/inappropriateness? | | We added subheadings by condition as requested. | | We inserted subheadings (line 356) 'Cancer', (line 368) 'COPD' and (line 384): 'Dementia') |
| 2.13 On page 21, please define ‘NOAC’. | | We changed the text accordingly. | | We changed Line 387 to: “with dispension of Novel Oral Anticoagulants (NOAC's) or …” |
| 2.14 In figure 1,2 and 3, I would suggest including a y-axis title. Is the risk-adjusted score what is plotted? | | We agree this could be presented more clearly. To accommodate the reviewer's suggestion, we added a description to Fig 1, 2 and 3, clarifying what is presented. | | We changed the description of Fig 1, 2 and 3. |
| 2.15 I found the ranking order of the relative increase in table 2 confusing. It is difficult to follow why ICU admissions are at the bottom of the list for COPD and fifth in the list for dementia for instance. I can see that you considered the fact that ICU admissions decreased for dementia patients a ‘positive’ thing and the fact that they increased for COPD patients a ‘negative’ thing. But the column is named ‘relative increase’ and therefore is confusing having + and – changes not in a more ‘intuitive’ order. I would suggest grouping them by ‘appropriateness/inappropriateness’ first, or to be clearer about which indicators are considered appropriate/inappropriate care. | | We changed the order of the quality indicators by splitting them up in indicators of appropriate or inappropriate care, showing them separately for each disease group. | | See changes table 2, below line 398. |
| 2.16 Discussion  In the first sentence, I would say ‘…trends in the appropriateness or inappropriateness of …’ or ‘quality indicators’. Otherwise, it sounds as the increase in the percentage of people admitted to an emergency department is appropriate. | | We changed the text accordingly. | | We changed line 405-406 to: “… three trends in the appropriateness and inappropriateness of end-of-life care …” |
| 2.17 I think you can discuss further the increasing number of contacts with GPs. Several reasons might explain these findings and also they might be influenced by the health care system context. Are there any major changes in health care provision in Belgium for instance that might explain these changes, particularly among dementia patients? An increase in health complexity could also explain higher primary care use. An increase in the number of contacts with GPs is an indirect way of evaluating quality and not necessarily would be considered more ‘appropriate’ in some contexts. It could also be reflecting a lack of coordination or a decrease in the level of continuity of care experienced by patients in primary care. You acknowledge in the limitations the fact that important aspects of the inappropriateness of care are not measured, as this is true not just for contacts with GPs but for all indicators. However, since changes in GP contacts have such an important relative increase, I found it important to discuss it a bit more in this section. | | Thank you for this remark, we agree the clear increase in GP contacts is a point that could be discussed further. Several possible reasons for this increase come to mind: between 2010 and 2015, the use of electronic health records in primary care practices has risen in Belgium, due to government financial support.^4^ This may have lead to an increasingly accurate registration and improved capture of GP contacts in administrative databases. Other changes have also affected primary care in this time period, including administrative reorganizations and the increasing emergence of group practices. As it stands, however, the connection between these events and our results are based on speculation. Further research is required to study this evolution. We added a sentence in the manuscript to support this. | | We added line 466-470: “General evolutions in the organization of primary care and the increased use of electronic health records in primary care might have also contributed to this increase, though further research is required to determine the contribution of these factor.” |
| 2.18 I think it would be important to emphasise in the discussion the fact that the indicators used, although recognised as important to evaluate the quality of care, should be understood as a population-level measure. You would not expect the proportion of people with ED admissions for instance to be reduced to 0, particularly considering end-of-life quality indicators are generally measured retrospectively, and from a clinical point of view it might not be easy to identify when a person is in the last 30 days or the last 180 days before death. That’s another good reason why examining the trend is important, as it gives context to the indicator. | | Thank you for this remark, we agree with this. We discussed the interpretation of the sets of quality indicators more in depth in previous publications.^5–7^ We added a sentence in the methods section to aid the reader towards correct interpretation. | | Line 248-251, we added: “Being population-level quality indicators, these indicators are not used to assess the quality of care of individual patients, nor do they imply the aim to be reduced to 0% (for inappropriate care) or 100% (for appropriate care).” |
| 2.19 I would be careful with the sentence ‘The increased use of specialized palliative care does not come with a reduction in inappropriate end-of-life care’, as it could be misinterpreted. The measures for each indicator are a population-based measure and therefore, if someone interprets that sentence at an individual level will be falling into an ecological fallacy. | | Thank you for pointing this out, we changed the text accordingly. | | We changed lines 511-513 to: “The increased use of specialized palliative care does not come with a population-level reduction in inappropriate end-of-life care.” |
| 2.20 When you say ‘To increase the appropriateness of end-of-life care, palliative care should be initiated earlier…’ it reads to me as if you were suggesting a specific intervention. As I mentioned above, from my point of view the focus of the conclusions should be put on indicators governments should be prioritising, based on their trend, rather than the interventions to do so. There might be different strategies to reduce the number of ED visits for instance. | | We agree and changed the manuscript accordingly. | | We changed lines 538-540: “To increase the appropriateness of end-of-life care, policy makers and practitioners should focus on earlier initiation of palliative care, while unnecessary care transitions should be avoided and unnecessary medication should be discontinued.” |
| 2.21 I think you should give a brief discussion regarding the generalisability of the results. | | We agree this is useful to discuss.  We therefore added a sentence about the generalizability of the results to the discussion section. | | We added lines 432-434:  “Finally, the data represent the full Belgian population in 2010-2015 and therefore provide generalizable information on the entire population’s health care use.” |
| **Reviewer #3** | | | | |
| 3.1 This a great piece of work demonstrating how population-level quality indicators can be used to understood the care provided to people approaching the end of life. It has important policy and practice implications for reviewing trends over time and making decisions for quality improvement and resource allocation. Risk adjusting the trends also demonstrates authors dedication to ensuring that they are not falsely claiming any changes which may be attributable to anything else. It is a very straightforward paper, easy to read accompanied with great figures, and well-written. This paper will be of interest of readers of this journal and a great contribution to the field. The authors can do this huge piece of work more justice by not only focusing on a select few changes that happened over the years, but by also discussing if no change for many others quality indicators is a good thing or not, referring to standards expected at a population level and what it might mean for healthcare and policy makers when a change of a certain magnitude is observed. | | Thank you for reviewing our manuscript. | | N/A |
| Major comments:  3.2 What's unclear from this paper is what constitutes a "large change". Are you only referring to changes >= 1% as large. this should be clearer in your methods. Additionally, please reflect on why this cut-off is meaningful or significant compared to a change of 0.5% over the years. While I appreciate that the second objective of the study was to identify QIs where the largest changes were observed, where there any QIs which should have improved yet remained stable over the years? It is a shame to include 70 QIs only focus on few, without any reflection or hypothesis around what changes you would expect see to based on the policies etc in place over time. | | We did not take a specific cut-off for a large change or small change. We chose to report both Relative and absolute increase in indicator score, (see table 2) as both are essential to judge whether a change is meaningful, so the readers have full information about the increases we determine as ‘large’ or ‘small’ increases.  While we focus only on a few indicators in the discussion and we agree further discussion on expected changes would be very interesting, we lack the space to do so in this publication, instead opting to focus on several surprising changes and providing all information to the reader to answer specific questions they might have. | | N/A |
| 3.3 In relation to the comment above, it would be good to remind the readers about the bench-marking values (Maybe in the Supplementary Files?). What is the expected standard for those QIs. This would make interpretations of the trend for each QI more meaningful. I appreciate that these values might be difficult to determine yet difficult for a reader to understand where things may need improvement without a benchmark. | | At line 525 till 528, we refer to the previously established relative standards: “Systematic monitoring of quality indicator scores and reestablishing relative standards, could help define goals for future improvement of the quality of end-of-life care.”  We added a reference to our previous work, for further reading.^5,7^ | | We added a reference in line 528. |
| 3.4 Discussion, especially the interpretation section of this manuscript seems a bit rushed. It focuses mainly on people with dementia and simply brushes over findings regarding the care of people with COPD and cancer. Please revise this section to reflect both overall and disease-specific interpretations of the findings. | | Our most important findings for people with COPD or cancer overlap with the findings for the general population. (overall increase in the percentage of people receiving specialized palliative care, especially in COPD and increase in the percentage of people with increasing contacts with a general practitioner. To be concise, we presented these together, instead of separately for each disease.  We did add a sentence to the general interpretation section of the discussion, discussing more about the increase in GP contact. (lines 390-392) | | (see also comment 2.17) |
| 3.5 Interpretation para 2 - Could it be also because of palliative care involvement, things which may have not been picked up previously are noticed and hence people end up in the ED? People with dementia have complex care needs. How can we be sure that all ED visits are inappropriate? If there are no community alternatives to replace the care provided in the ED, what should happen? If there are alternatives providing very similar services in Belgium, please reflect on this. | | Thank you for these interesting questions. Although we agree that it is possible that the involvement of palliative care would lead to more ED visits, especially if there would be no community alternatives to replace the care provided in the ED, we think this is unlikely. Another study performed by the authors has in fact shown the exact opposite.^8^ It demonstrated that people who received palliative home care support had, compared with those who did not, a lower risk of ED admission (15.2%vs28.1%; RR=0.54, 95% CI 0.51 to 0.57). Moreover, in Belgium there are many community alternatives for the care provided in the ED. We therefore argue that it is more likely that the increase in palliative care and the increase in ED visits are two similar, but independent trends. | | We added a reference to the study about the impact of palliative care on resource use to the text. (line 485) |
| 3.6 Interpretation para 3 - Some barriers to de-prescription are stated. Are these barriers faced even after using a review tool such as STOPPFrail? Please reflect on further implications of polypharmacy, multimorbidities and ED attendances. These are likely to be interconnected rather than separate. | | Some of the barriers might indeed be removed by using a review tool such as STOPPFrail. However, the authors are currently performing a qualitative study on deprescribing and found that the STOPPFrail tool is hardly known are used in clinical practice in Belgium. Although the use of the STOPPFrail tool could facilitate deprescribing, that alone is probably not sufficient to mitigate all barriers to de-prescribing. Patients and family members should also be involved in the decision-making process and they may be hesitant to discontinue long-term preventive medication. Hence, using a medication review tool such as STOPPFrail will probably not mitigate all barriers to de-prescribing. However, discussing this further in the paper would lead us too far from the study objectives, but we fully agree this should be investigated further. | | N/A |
| 3.7 Implications - "to increase appropriateness of end-of-life care, an effort should be made to reduce the number of ED and ICU admissions of people with serious and chronic illness near the end of life. Agreed that this is important but why are people having these admissions? Surely, this is a research question for future research/direction. Also this is oversimplyfing the situation and saying that if we reduce these admissions, we will increase the provision of appropriateness of EOL care. | | Thank you for this question. We agree it is an interesting research question for future research. We did not mean to underestimate the complexity of the issue: reducing the number of ED and ICU admissions implies taking on the causes of these admissions (when possibly inappropriate), for example by preventively addressing sudden increase in symptom burden that cannot be managed at home or in a home-replacing setting.^6^  We have therefore changed these sentences in the discussion. | | We changed lines 514-522 to: “We suggest that, to increase appropriateness of end-of-life care, an effort should be made to look for the causes of ED and ICU admissions of people with serious and chronic illness near the end of life, to address these causes, and to increase the availability of community alternatives such as palliative home care available to meet these people’s complex care needs." |
| 3.8 Authors should acknowledge that it is difficult to understand whether the care was appropriate or not by simply using QIs. QIs are very useful for understanding population-level care and their use here is correct, but this is a limitation. | | We added a sentence to clarify this in the methods section. (see also comment 2.18) | | (see comment 2.18) |
| 3.9 There is some mention of policy and attitude shift in Belgium around the importance of palliative care and a statement around an improvement in trends might be expected. Bringing in more context here and in the Discussion might be helpful. This would also make it easier to understand the findings' implications on future policy and quality improvement. | | We agree bringing in this context in the discussion is helpful. We added a sentence accordingly. | | We added lines 455-458:  "This seems to imply the increasing emphasis on appropriateness of end-of-life care in Belgian government advisory documents between 2009 and 2017(26,27) coincides with an increased use of specialized palliative care." |
| Minor comments:  3.10  There are few typos here and there (health care and healthcare, 1ste Jan etc.) - please have a look at these. | | We have corrected the typos. | | Corrected several typos. |
| 3.11 Your title can be simplified to "Trends in appropriateness of end-of-life care in people with cancer, COPD or with dementia measured with population-level quality indicators". This is just a suggestion because "Do quality indicators improve over time" does not really make sense. "Does quality of care improve over time" makes more sense, but one QIs are only one part of measuring and understanding the quality of care. | | Thank you for these suggestions. We changed the title of the manuscript to: "Trends in appropriateness of end-of-life care in people with cancer, COPD or with dementia measured with population-level quality indicators". | | We changed the manuscript title. |
| 3.12 Introduction: "These efforts in research and policy might be a reflection of a broader societal evolution towards more attention for appropriateness of end-of-life care and positive attitudes towards palliative care." Please back this up with a reference. | | Since it was our own hypothesis that the efforts in research and policy could be a reflection a broader societal evolution, we cannot back this statement up with a reference. We therefore decided to delete this sentence and rewrote the following one. | | We changed lines 130-131 to:  “Hence, we could expect a shift towards more appropriate end-of-life care.” |
| 3.13 The first objective of the study reads as not a trend analysis of change over years but a comparison between 2010 and 2015. The second objective sounds grammatically incorrect. Could you please re-word these? | | Thank you for this remark. We have reworded both research questions. | | Lines 145-151 we changed to: “1. Did the appropriateness of end-of-life care for people dying from cancer or COPD or with dementia change between 2010 and 2015 in Belgium?  2. What indicators of appropriate or inappropriate end-of-life care have had the largest changes in this time period?” |
| 3.14 Could you please add the percentages for "being officially recognized as having high care needs" and "being entitled to a higher degree of reimbursement due to lower degree of self-reliance" in the descriptive tables as they were also entered into regression model for risk adjustment? | | Unfortunately, at the moment of this revision, we no longer have access to the original data, due to ethical and privacy reasons, so we cannot add the requested numbers. | | N/A |
| 3.15 Please add to your tables that you are reporting percentages. | | We would like refer the reviewer to our answer to comment 2.11 from reviewer 2. | | (see comment 2.11) |
| 3.16 In the results section, when referring to disease specific trends, please only refer to the relevant figure. | | We adjusted this in the revised manuscript. | | We changed lines 361, 366 and 376. |
| 3.17 Table 2 - Think carefully about what you'd like the readers to get from this table. It is a busy and hard to read table, which actually contains valuable information. If you would like readers to evaluate each disease by themselves, please list the improvement scores for each disease group one after other rather than side by side. If you would like readers to easily compare some of the same QIs across disease (e.g. ED admissions, palliative care involvement), please list the QIs common for all groups first, then the others side by side. Either way, this table needs to be revised. | | We agree with the reviewer that Table 2 is difficult to read. This was also mentioned by reviewer 2. We would therefore like to refer the reviewer to our answer to comment 2.15 of reviewer 2. | | (see comment 2.15) |
| 3.18 The first limitation is about relying on death certificate data to select the COPD and dementia populations but there is no references or explanations about the COPD population's identification. | | We would like to refer the reviewer to the paragraph about the selection of the population in the methods section. In that paragraph we explain for the three disease groups how the populations were identified, including the COPD population.  Lines 186-198:  “People dying from cancer (ICD10 codes C00-C99) or COPD (ICD10 codes J41-J44) were identified using the underlying cause of death on the death certificate.” | | N/A |
| 3.19 Interpretation Para 2 - People with dementia near the end of life in Belgium often live in a nursing home. - Please provide a reference or give the percentage from your findings. | | Thank you for this remark, we added a reference to Reyniers et al, 2015.^9^ | | We added a reference. |
| 3.20 Figures - Please use the x-axis used in Figure 2, INDICATORS OF INAPPROPRIATE GENERAL TREATMENT AND MEDICATION. for all other figures as this is the most readable one. | | Thank you for noticing, we changed all the year-indications on the X-axis of figures 1, 2 and 3 to the same settings, to increase readability. | | We changed the lay-out of the X-axis of figures 1, 2 and 3. |
| 3.21 Supplementary Files - Typo - secundary and please state for net taxable income quintiles 1-5 which one indicates the highest vs lowest. | | We corrected the typo and we indicated the highest and lowest quintiles for net taxable income. | | We changed the typo and net tax income in Supplementary tables 1, 2 and 3. |

**References**

1.	Murray SA, Kendall M, Boyd K, Sheikh A. Illness trajectories and palliative care. BMJ. 2005;330:1007–11.

2.	De Schreye R, Houttekier D, Deliens L, Cohen J. Developing indicators of appropriate and inappropriate end-of-life care in people with Alzheimer’s disease, cancer or chronic obstructive pulmonary disease for population-level administrative databases: A RAND/UCLA appropriateness study. Palliat Med. 2017;31:932–45.

3.	Bao J, Chua K-C, Prina M, Prince M. Multimorbidity and care dependence in older adults: a longitudinal analysis of findings from the 10/66 study. BMC Public Health. 2019;19:585.

4.	Geïntegreerde praktijkpremie | Domus Medica [Internet]. [cited 2021 Dec 20]. Available from: https://www.domusmedica.be/expertisedomein/ict/geintegreerde-praktijkpremie

5.	De Schreye R, Smets T, Deliens L, Annemans L, Gielen B, Cohen J. Appropriateness of End-of-Life Care in People Dying From COPD. Applying Quality Indicators on Linked Administrative Databases. J Pain Symptom Manage. 2018;56:541-550.e6.

6.	De Schreye R, Smets T, Annemans L, Deliens L, Gielen B, De Gendt C, et al. Applying Quality Indicators For Administrative Databases To Evaluate End-Of-Life Care For Cancer Patients In Belgium. Health Aff (Millwood). 2017;36:1234–43.

7.	De Schreye R, Smets T, Deliens L, Annemans L, Gielen B, Cohen J. Appropriateness of End-of-Life Care in People Dying With Dementia: Applying Quality Indicators on Linked Administrative Databases. J Am Med Dir Assoc. 2020;

8.	Maetens A, Beernaert K, De Schreye R, Faes K, Annemans L, Pardon K, et al. Impact of palliative home care support on the quality and costs of care at the end of life: a population-level matched cohort study. BMJ Open. 2019;9:e025180.

9.	Reyniers T, Deliens L, Pasman HR, Morin L, Addington-Hall J, Frova L, et al. International Variation in Place of Death of Older People Who Died From Dementia in 14 European and non-European Countries. Journal of the American Medical Directors Association. 2015;16:165–71.
