# Supplementary material for: Trends in appropriateness of end-of-life care in people with cancer, COPD or with dementia measured with population-level quality indicators
Source: PLoS One. 2023 Feb 1;18(2):e0273997. doi: 10.1371/journal.pone.0273997 (PMC9891500; doi:10.1371/journal.pone.0273997)
Supplement: Attachment — Submitted filename: Response to Reviewers.docx [file pone.0273997.s009.docx]

Trends in appropriateness of end-of-life care in people with cancer, COPD or with dementia measured with population-level quality indicators

**PONE-D-21-14989 - Reply to editor's comments**

This document lists all editor's responses and comments, with answers and changes to the text, if applicable. All line numbers are referring to the manuscript with tracked changes.

| **Comments by the Academic editor** | | |
| --- | --- | --- |
| **Editor comment** | **Response** | **Changes to the manuscript** |
| 1. Abstract - the results and conclusions could be strengthened. You have important novel findings that could be better conveyed in the abstract. You need to reduce words in the preceding sections to increase reporting in the results. Reduce words in the intro - this is too long. Remove the years from the aim, this is stated in the design, and just state Design: A population-level ....Participants: All people who died.... Removing we conducted - it is not needed. | We reduced the number of words in the introduction. We removed the years from the aim and changed the design as suggested. | We changed the introduction of the abstract (lines 28-35) to "*Measuring changes in the appropriateness of end-of-life care provided to patients with advanced illness such as cancer, COPD or dementia can help governments and practitioners improve service delivery and quality of life. However, an assessment of a possible shift in appropriateness of end-of-life care across the population is lacking*."  We adopted all other suggested changes (lines 36-49) as they are into the manuscript. |
| 2. Results - We identified three main trends overtime across the three disease groups of increasing use of: family physician in the last 30 days of life (give +% for each group); receipt of specialist palliative care mostly initiated in the last 14 days of life (give +% for each ; and use of emergency department in the last 30 days of life (give +% for each). Remove sentence re additional trends as not giving any information - or keep and give examples of these trends | We added the percentage for each group, as suggested. We removed the suggested sentence.  Additionally, we removed 6 unnecessary words to reduce the abstract word count. | We changed the results of the abstract (lines 50-62) to: "*We identified three main trends over time across the three disease groups of increasing use of: family physicians in the last 30 days of life (+21.7% in cancer, +33.7% in COPD and +89.4% in dementia); specialist palliative care in the last 14 days of life (+4.6% in cancer, +36.9% in COPD, +17.8% in dementia); and emergency department in the last 30 days of life (+7.0% in cancer, +4.4% in COPD and +8.2% in dementia)*." |
| 3. Conclusions - don't really link to your results. Your conclusion in the manuscript is much stronger and clearer. Can you review and revise detailing e.g. identification of appropriate care xx and inappropriate to answer your stated aim, then focus for policy makers. | We reviewed the conclusion as suggested.  Additionally, based on this change, we also reviewed the conclusion of the main text and made minor changes. | We changed the text of the conclusion of the abstract (lines 64-75) to "*Although we found an increase of both specialized palliative care and generalist palliative care use, we also found an increase in potentially inappropriate care, including ED and ICU admissions. To increase the quality of end-of-life care, both timely initiating (generalist and specialist) palliative care and avoiding potentially inappropriate care transitions, treatments and medications need to be quality performance targets."*  We changed the text of the conclusion (lines 489-492) to*: "To increase the appropriateness of end-of-life care, policy makers and practitioners should focus both on timely initiation of palliative care and on avoiding or discontinuing potentially inappropriate care transitions, treatments and medication."* |
| 4. Introduction - Earle et al 2008 reference reporting data > 30 years old. I can see that important to have reference re use of chemo, but there must be a more recent reference given high income countries typically monitor use of chemo in last 30 days of life. Can you review and update. it is a bit distracting to use such old data when you are arguing about more recent change | We update the reference with more recent literature and changed the text accordingly. | We changed the text in lines 97-102 to " *Pacetti et al., for instance, found high chemotherapy use during the last 4 weeks of life, with the individual clinician being the sole predictor of continuation of chemotherapy between 2010 and 2012."*(1) |
| Use of language - terms of handicap and elderly people are not generally used as considered derogatory. Can you remove - this is line 245, and state judged by the GP for a person with multiple complex needs - you could give examples polypharmy, high level of disability, multimorbidity. FYI see useful publication on language - Lundebjerg NE, Trucil DE, Hammond EC, Applegate WB. When It Comes to Older Adults, Language Matters: Journal of the American Geriatrics Society Adopts Modified American Medical Association Style. Journal of the American Geriatrics Society 2017; 65(7): 1386-8. | We removed the sentence on line 264, as it did not provide any additional information to the reader. | We removed the sentence on line 264, as it did not provide any additional information to the reader. |
| • Results - table 1 - state Age mean (SD) - you need to report the (SD) for each mean given - as standard reporting 68.2 (insert SD). This is important for the interpretation of the data presented | We added the SD for each mean age provided in the table, as requested. | (see changes in table 1) |

**References**

1. Pacetti P, Paganini G, Orlandi M, Mambrini A, Pennucci MC, Del Freo A, et al. Chemotherapy in the last 30 days of life of advanced cancer patients. Support Care Cancer [Internet]. 2015 Apr; Available from: http://www.ncbi.nlm.nih.gov/pubmed/25900109
