# Supplementary material for: Trends in appropriateness of end-of-life care in people with cancer, COPD or with dementia measured with population-level quality indicators
Source: PLoS One. 2023 Feb 1;18(2):e0273997. doi: 10.1371/journal.pone.0273997 (PMC9891500; doi:10.1371/journal.pone.0273997)
Supplement: Attachment — Submitted filename: Response to Reviewers.docx [file pone.0273997.s010.docx]

Trends in appropriateness of end-of-life care in people with cancer, COPD or with dementia measured with population-level quality indicators

**PONE-D-21-14989 - Reply to editor's comments**

This document lists all editor's responses and comments, with answers and changes to the text, if applicable. All line numbers are referring to the manuscript with tracked changes.

| **Comments by the Academic editor** | | |
| --- | --- | --- |
| **Editor comment** | **Response** | **Changes to the manuscript** |
| 0. Thank you for the opportunity to review the manuscript titled “Trends in appropriateness of end-of-life care in people with cancer, COPD or with dementia measured with population-level administrative data: do quality indicators improve over time?” The authors observed mixed trends of end-of-life care in people dying from cancer, COPD or with dementia using indicators of appropriateness and inappropriateness of end-of-life care. This manuscript is well written. However, I have a few minor concerns about the methods and would like some clarifications. | Thank you for taking the time to assess our manuscript. | N/A |
| 1. Introduction: the authors mentioned that “Earle et al., for instance, found an increase in chemotherapy use and continuation of chemotherapy closer to death in Medicare patients with cancer in the US between 1993 and 1996.10 ” However this evidence applies to the trend in chemotherapy more than 20 years ago. I would suggest that the authors should use more recent references. | Apparently something went wrong with the version that was provided to the editor. In the current version, the text the editor refers to is no longer included in the manuscript. In the last response to the reviewers, following a similar question, we updated the reference with more recent literature and changed the text accordingly. | Last version, we changed the text in lines 97-102 to " *Pacetti et al., for instance, found high chemotherapy use during the last 4 weeks of life, with the individual clinician being the sole predictor of continuation of chemotherapy between 2010 and 2012."*(1)  We did not make any new changes. |
| 2. Methods: It seems the analytic cohort only consists of the decedents whose health care use was reimbursed. Despite the authors acknowledged the limitation in the paper, it would be helpful to describe the proportion of reimbursed care and non-reimbursement care, so that readers can have a general sense about how representative the analytic sample is. | Subscription to health insurance is legally mandatory in Belgium for all citizens. As such, the cohort consists of almost the full population. This is mentioned on lines 142-144 of the methods section.  The limitation mentioned in the discussion is about types of care that are not reimbursed, rather than people not being included in the study. This is explained in lines 427-430 of the discussion. | N/A |
| 3. Method: The risk-adjusted score was calculated for each year. However, only the risk adjusted scores of 2010 and 2015 were used to calculate the increase and decrease. This is certainly not trend analysis, with the information between these two years all missing. I would suggest a trend test by incorporating the adjusted indicator scores of year 2011-2014. | We agree with the editor: the calculation of the score differences between 2010 and 2015 was an addition to and not part of the trend analysis.  This is described in lines 261 – 270 of the methods section. We aimed to make the distinction by writing: “This way, a trend analysis was performed, (…).We also calculated all risk adjusted indicator score differences between 2010 and 2015.” We hope this clarifies these are two distinct steps.  The results of the trend analysis are depicted in figures 1, 2 and 3, while the results of the difference calculation is shown in table 2. | N/A |
| 4. Discussion: the authors observed an increase in palliative care while an increase in ED visits and ICU admission too. The discussion about the contradicting trends should be strengthened, i.e. what is the possible reason? Is there any policy change during the study period? | During the study period, there were no policy changes that could explain the increases. We agree with the editor this should be made more clear in the text.  We also agree the argument by Teno et al, corroborated by our findings, should be presented more strongly.  We changed the text accordingly. | Lines 434-435 added: “*Between 2010 and 2015, no relevant policy changes that could explain this evolution were observed.*”  Lines 436-437 changed to: “*This suggests that palliative care is often combined with aggressive treatment rather than replacing it*.” |

**References**

1. Pacetti P, Paganini G, Orlandi M, Mambrini A, Pennucci MC, Del Freo A, et al. Chemotherapy in the last 30 days of life of advanced cancer patients. Support Care Cancer [Internet]. 2015 Apr; Available from: http://www.ncbi.nlm.nih.gov/pubmed/25900109
